# StreptomeDB 2.0—an extended resource of natural products produced by streptomycetes

**DOI:** 10.1093/nar/gkv1319

**Published:** 2015-11-28

**Authors:** Dennis Klementz, Kersten Döring, Xavier Lucas, Kiran K. Telukunta, Anika Erxleben, Denise Deubel, Astrid Erber, Irene Santillana, Oliver S. Thomas, Andreas Bechthold, Stefan Günther

**Affiliations:** 1 Pharmaceutical Bioinformatics, Institute of Pharmaceutical Sciences, Albert-Ludwigs-University, Hermann-Herder-Strasse 9, Freiburg 79104, Germany; 2 School of Life Sciences, Division of Biological Chemistry and Drug Discovery, University of Dundee, James Black Centre, Dow Street, Dundee DD1 5EH, UK; 3 Chair for Bioinformatics, Department of Computer Science, University of Freiburg, Georges-Koehler-Allee 106, Freiburg 79110, Germany; 4 Pharmaceutical Biology, Institute of Pharmaceutical Sciences, Albert-Ludwigs-University, 79104 Freiburg, Germany

## Abstract

Over the last decades, the genus *Streptomyces* has stirred huge interest in the scientific community as a source of bioactive compounds. The majority of all known antibiotics is isolated from these bacterial strains, as well as a variety of other drugs such as antitumor agents, immunosuppressants and antifungals. To the best of our knowledge, StreptomeDB was the first database focusing on compounds produced by streptomycetes. The new version presented herein represents a major step forward: its content has been increased to over 4000 compounds and more than 2500 host organisms. In addition, we have extended the background information and included hundreds of new manually curated references to literature. The latest update features a unique scaffold-based navigation system, which enables the exploration of the chemical diversity of StreptomeDB on a structural basis. We have included a phylogenetic tree, based on 16S rRNA sequences, which comprises more than two-thirds of the included host organisms. It enables visualizing the frequency, appearance, and persistence of compounds and scaffolds in an evolutionary context. Additionally, we have included predicted MS- and NMR-spectra of thousands of compounds for assignment of experimental data. The database is freely accessible via http://www.pharmaceutical-bioinformatics.org/streptomedb.

## INTRODUCTION

The growing number of annotated natural products (NPs) in databases, such as Super Natural II (∼326 000 compounds; 2D structures; physicochemical properties; predicted toxicity class; potential vendors) (1), KNApSAcK (∼51 000 metabolites; ∼111 000 metabolite-species pairs) (2), UNPD (∼229 000 compounds; 3D structures) (3) or NORINE (∼1200 NRPs) (4) reflects the increasing interest in these molecules. StreptomeDB is, to the best of our knowledge, the biggest compilation of NPs produced by streptomycetes. Its content has been collected from thousands of abstracts and full papers by text mining methods and extensive manual curation. In addition, it includes comprehensive background information such as host organisms, predicted physicochemical properties, synthesis routes and biological activities.

*Streptomyces* is a genus of Gram-positive actinobacteria. They can be found all around the world in soil samples and successfully inhabit many terrestrial and aquatic niches (5). Since the discovery of streptomycin by Albert Schatz in the group of Selman Waksman in 1943 (6), they have become one of the best studied bacterial *genera*. Over 60% of all known antibiotics have been isolated from streptomycetes (5). Many of these compounds are approved drugs, such as the well-known agents tetracycline, daptomycin and chloramphenicol (7). Besides antibiotics, there is a rich diversity of other secondary metabolites with a plethora of different biological activities and therapeutic potential exclusively produced by streptomycetes. Blockbuster drugs like the anti-parasitic agent avermectin, the immunosuppressant rapamycin or the lipase inhibitor lipstatin are just a few examples (8,9,10). There is also a large number of potential antibiotics or other bioactive compounds found in StreptomeDB which are not in clinical use (11). As the recent development of highly effective griselimycin analogs shows (12), these molecules can be considered as possible cornerstones for the future development of semi-synthetic drugs (13).

A common starting point of a modern drug discovery campaign is an *in silico* high-throughput screening for small molecules or fragments that can interact with a therapeutic target of interest (14). The method strongly relies on high quality and diverse compound libraries like StreptomeDB. Although NPs are in many cases harder to obtain than compounds produced by chemical synthesis and are rarely found in vendors’ catalogs, they are nonetheless a valuable addition to screening libraries (11,13). Due to their complex stereochemistry, they are often more selective than synthetic compounds and are particularly suitable–for addressing low-druggable targets (15,16). Furthermore, NPs are in most cases produced enantiomerically pure, whereas enantiomeric purification of synthetic compounds can be an expensive and exhausting task (17).

Due to the variety of chemical precursors and diversity of produced scaffolds, streptomycetes are versatile hosts for industrial heterologous expression (18). Developments in biotechnology and synthetic biology have led to artificially minimized host strains that can be utilized for the production of various compounds (19). For example, the genetically engineered SUKA strains, descendants of the industrial microorganism *Streptomyces avermitilis*, which is already known for its highly efficient production of the anthelmintic agent avermectin (20), have proven useful as a heterologous host in the production of several secondary metabolites. Beyond the production of bioactive compounds, the rich diversity of chemical scaffolds produced by streptomycetes enables their use as precursors for many semi-synthetic approaches. For example, *Streptomyces venezuelae* has recently been employed as a producer of bisabolenes (21), which are sesquiterpenes present in several essential oils in plants. The advanced biofuel bisabolane (22) is synthesized from them in a single step by chemical hydrogenation (23).

Although StreptomeDB demonstrates that there are already thousands of compounds isolated from streptomycetes, their potential as a source of NPs is far from exhausted (24). Advancing methods still allow for the discovery of new strains and compounds, such as the phenalinolactones, i.e. terpene glycosides with a rare, highly oxidized γ-butyrolactone structure (25).

The broad interest in streptomycetes has so far led to more than 23 000 publications in PubMed. In the last years, the number of publications per year was constantly growing to reach about 1000 in 2014, stressing the need for an updated StreptomeDB that makes this vast amount of data accessible.

**Figure 1.**
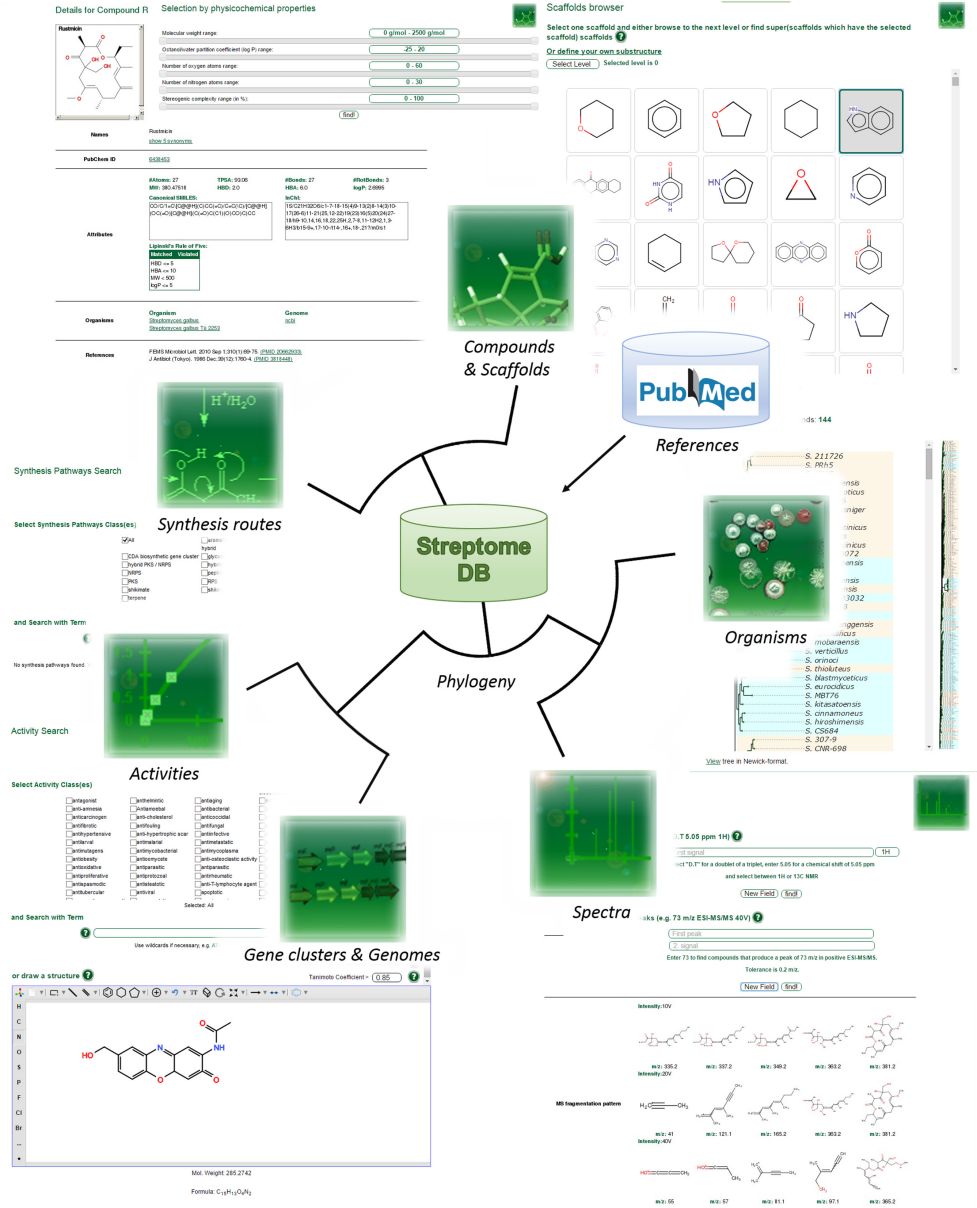
Features and search options implemented in StreptomeDB.

Here we present StreptomeDB 2.0, a major update with an increase from about 2500 to over 4000 compounds, hundreds of which required manual drawing prior to insertion into the database, and more than 2500 host strains. Additionally, the introduced new features assist in the navigation through the vast chemical diversity produced by streptomycetes (Figure 1). This allows for a deeper understanding of the complexity and evolution of the synthesis machinery that makes this *genus* so attractive for academic and industrial research.

## MATERIALS AND METHODS

### Data collection

Manual curation of literature was carried out following the protocol of the original database (11). For the sake of completeness, special focus was put on gathering compounds from full texts. Additionally, hundreds of compounds with name not provided in the referenced manuscript or not found in PubChem were manually depicted prior to insertion into the database.

### Generation of the phylogenetic tree

The phylogenetic tree is based on the most common 16S rRNA sequence per strain. Over 20 000 sequences from freely available sources, such as silva (26) and ENA (27), were filtered for integrity and length and mapped to organisms in StreptomeDB. This resulted, in some cases, in over 150 different 16S rRNA sequences for a single strain. If it was not possible to identify the best sequence, a hypothetical, most common sequence was derived. For this approach, the strain-specific datasets were first scanned with BLAST+ (28) for the sequences that produce the most hits with an *E*-value below 1 × 10^−4^ and a coverage over 97%. If this resulted in more than one sequence, a consensus sequence was built with the dumb_consensus function, as implemented in Biopython (29). Sequences were aligned with ClustalW (30). Phylogenetic analysis of the resulting alignment was performed with the MEGA software package (31) using the maximum likelihood and maximum parsimony algorithms, each with 250 bootstrap replications. Sub-clusters in the tree were detected by summarizing all leafs that have a common ancestor node within range of 0.07 nt substitutions per side, using the DendroPy library (32). The final editing and visualization was done with the ETE 2 toolkit (33).

### Gene clusters, genomes and taxonomy

If available, links to gene clusters in DoBISCUIT (34) or MiBIG(35) are provided in StreptomeDB. Host organisms are linked to freely available full genomes from GenBank (36) and entries in the NCBI taxonomy database (37). Substrains and mutants without taxonomy ID are linked to their primary strains.

### Scaffold-based molecular decomposition

The Scaffold Decomposition tool included in Canvas 2.3 (Schrödinger, LLC, New York, NY, USA) was used to process molecules from StreptomeDB and extract all represented scaffolds. Small molecules can comprise one or more level 0 scaffolds, i.e. root cyclic or polycyclic independent structures. Two chemically equivalent or different level 0 entities connected by an aliphatic or functionalized linker define a level 1 scaffold. Subunits composed of a level 0 plus a level 1 scaffold generate a level 2 scaffold, and so on (38).

### Prediction of fragmentation patterns in mass spectra

The software CFM-ID, based on competitive fragmentation modeling to produce a probabilistic generative model for electrospray tandem spectrometry (ESI-MS/MS) fragmentation (39), was used to predict the fragmentation spectrum of molecules with ≤30 heavy atoms in positive ionization mode at 10, 20 and 40 V, and assign the resulting peaks to their chemical structure. For each intensity level, the most intense peaks (max. 5) are reported in StreptomeDB.

### Prediction of ^1^H and ^13^C NMR peaks

The nuclear magnetic resonance (NMR) Predictor tool, as implemented in the command-line platform cxcalc (Marvin 15.4.13.0, 2015, ChemAxon, http://www.chemaxon.com), was used to predict the ^1^H and ^13^C NMR spectra of all molecules.

## NEW FEATURES AND UPDATES

### Phylogenetic tree

A new feature of StreptomeDB is the comprehensive phylogenetic tree based on 16S rRNA sequences. Considering the usually similar six or more 16S rRNA (40) sequences present in a *Streptomyces* genome (and in some cases several similar sequencing attempts for one sequence, which cannot be distinguished in quality or length), we generated a consensus sequence for each strain. We have chosen a tree based on 16S rRNA to achieve the highest possible coverage of the data contained in StreptomeDB. For a better overview and because of the relatively short evolutionary distance, more than 1200 sub strains are represented by 340 parent stains, e.g. clicking on *‘Streptomyces griseus’* leads to 128 compounds produced either by *S. griseus* itself or one of its 58 sub strains, such as *S. griseus* or *S. griseus var. psychrophilus*. The apparently small number of 340 shown strains comprises more than two-thirds of the compounds in the database, therefore indicating a good coverage of the available molecules. Additionally, it is possible to access the phylogenetic tree from any search, with the producing organisms of the related compounds highlighted. This provides the possibility to visualize the distribution of a certain scaffold, chemotype, compound, bioactivity or synthetic route in an evolutionary context.

### Scaffold-based navigation and search system

A new scaffold-based navigation has been integrated and the compound search system has been substantially improved. All molecules have been redefined by means of their scaffold framework (38), and there is now the possibility to browse through the gathered chemical scaffolds. At each scaffold level (*Search -> Scaffolds browser*), the user can choose one or several scaffolds, which are sorted by frequency among the gathered compounds, and display either compounds or higher-level scaffolds. A phylogenetic tree with highlighted producing organisms can now be easily accessed. Furthermore, compound cards contain a list of represented scaffolds, which in turn link to a list of compounds that contain the scaffold. This enables the bidirectional browsing through the chemical diversity of the database on a structural basis and its connection to the evolution of the producing organisms. Table 1 shows that StreptomeDB contains more than 1000 level 0 scaffolds and hundreds of higher-level scaffolds, including 281 scaffolds of very advanced framework and high molecular weight. In addition, searches based on user-defined chemical structure patterns can be performed taking advantage of fast substructure searches (Search -> Compound(structure) section).

**Table 1.** Chemical diversity of StreptomeDB

Scaffold level	No. of unique scaffolds	Scaffold level	No. of unique scaffolds
0	1,032	6	213
1	732	7	137
2	672	8	96
3	559	9	91
4	469	10	84
5	314	>10	281

Scaffold levels and the number of unique scaffolds included in the database.

### Additional content

Compound cards now provide predicted MS and NMR spectra for thousands of compounds. The MS/MS spectra include the five most intensive peaks for 10, 20 and 40 V energy levels. The chemical structure of each peak is depicted. NMR data are listed in a table that includes intensity, multiplicity and chemical shift for both, ^1^H and ^13^C NMR. It is possible to query the database for any combination of ^1^H and ^13^C signals or MS peaks. Additionally, there is the possibility to query the database by physicochemical properties, including molecular weight, octanol/water partition coefficient (*c*log *P*_o/w_), number of nitrogen and oxygen atoms and stereogenic complexity, i.e. C*/C_T_ (16). Finally, StreptomeDB includes links to all publicly available gene clusters and genomes for compounds and host organisms.

### Back end and library updates

All software libraries related to the web page, its database back end and the in-house curation platform were updated to the latest versions. The structures of curated molecules were mapped to canonical SMILES using ChemicalToolBoX (41). The update steps have been redesigned to upload new molecules and related data to the database back end automatically, based on the uniqueness of structures.

## CONCLUSION AND FUTURE PROSPECTS

Here we present a major update on StreptomeDB, which implements a series of new features to offer easier access to the huge amount of data related to streptomycetes, which is hidden in literature. Furthermore, StreptomeDB improves the integration of this information in a chemical or evolutionary context.

From a chemical point of view, the implementation of a comprehensive scaffold-based navigation system enables browsing the rich diversity of NPs produced by streptomycetes. Scaffold-based molecular representations are a prominent tool in medicinal chemistry (38) and allow for classifying and comparing the coverage and content of chemical libraries. We could identify hundreds of naturally occurring level 1 scaffolds not found in purchasable compounds (15). This is of particular interest in drug discovery, as on average each commercialized drug contains a novel scaffold, thus stressing the crucial relevance of mining both natural sources and literature for molecules. Clearly, secondary metabolites produced by streptomycetes are an attractive source of chemical diversity.

The phylogenetic tree enables the visualization of the distribution of these scaffolds and the compounds they represent. Especially in projects that deal with poorly studied strains, this feature offers an easy access to additional references and background information of closely related organisms. It is a new approach toward the understanding of the evolution of secondary metabolism. Together with the offered gene clusters and genomes it provides all building blocks for comparative genome analysis, reconstruction of metabolic networks, or building a customized, more specialized phylogenetic tree. Motivated by the decreasing costs and massive efforts that are currently undertaken in genome sequencing, the number of published genomes and gene clusters is quickly increasing (42). Therefore, we are looking forward to expanding these features to an even greater extent.

We have also improved the characterization and querying of compounds by including predicted MS and NMR data. These techniques are widely used in structural determination of secondary metabolites, and we realized that a tool for comparing experimental MS spectra with that of compounds in the database would be extremely beneficial for the users of StreptomeDB. Now this is possible by querying the database with experimental peaks, either from MS or NMR determination, to retrieve compounds with matching patterns.

Due to the increasing popularity of StreptomeDB, we want to encourage users to contact us if they find missing data or have optimization proposals.

In conclusion, StreptomeDB is a versatile platform for the gathering of information for projects that deal with streptomycetes. It stands out from other popular NP databases with a broader focus, such as Supernatural II, KNAPsACK or UNPD (1,2,3): built on top of a highly networked structure, StreptomeDB facilitates exposing coherences between different data, such as scaffolds, activities or phylogenetic distribution. It offers an ideal starting point and a vast amount of supplementary resources for the discovery of new compounds, virtual screening campaigns, biochemical engineering and cheminformatics.

## AVAILABILITY

StreptomeDB is freely available, open to all users, and has no login requirements. All compounds can be downloaded with metadata in SD-Format at http://www.pharmaceutical-bioinformatics.org/streptomedb/download.
